# Knowing antimicrobial resistance in practice: a multi-country qualitative study with human and animal healthcare professionals

**DOI:** 10.1080/16549716.2019.1599560

**Published:** 2019-07-11

**Authors:** Maddy Pearson, Clare Chandler

**Affiliations:** Department of Global Health and Development, London School of Hygiene and Tropical Medicine, London, UK

**Keywords:** Antimicrobial Resistance, Public health, antibiotic prescribing, antibiotic dispensing, antimicrobial resistance, healthcare professionals

## Abstract

**Background**: Antimicrobial resistance (AMR) is a growing global problem. Raising awareness is central to global and national action plans to address AMR in human and livestock sectors. Evidence on the best ways to reduce antibiotic use, and the impact of awareness raising activities is mixed. This paucity of evidence is acute in Low-Middle-Income Country (LMIC) settings, where healthcare professionals who prescribe and dispense antimicrobial medicines are often assumed to have limited awareness of AMR and limited knowledge of the optimum use of antimicrobials.

**Objectives**: This research aimed to explore AMR awareness among human and animal healthcare professionals and the contextual issues influencing the relationship between awareness and practices of antimicrobial prescribing and dispensing across different LMIC settings.

**Methods**: Qualitative interviews and field observations were undertaken in seven study sites in Ethiopia, India, Nigeria, the Philippines, Sierra Leone and Vietnam. Data included transcripts from interviews with 244 purposively sampled healthcare professionals, analysed for cross-cutting themes.

**Results**: AMR awareness was high among human and animal healthcare professionals. This awareness of AMR did not translate into reduced prescribing and dispensing; rather, it linked to the ready use of next-line antibiotics. Contextual factors that influenced prescribing and dispensing included antibiotic accessibility and affordability; lack of local antibiotic sensitivity information; concerns over hygiene and sanitation; and interaction with medical representatives.

**Conclusions**: The high awareness of AMR in our study populations did not translate into reduced antibiotic prescribing. Contextual factors such as improved infrastructure, information and regulation seem essential for reducing reliance on antibiotics.

## Background

Antimicrobial Resistance (AMR) is a global problem [1], rising in both biological prevalence and global health strategy prominence [2]. Resistance to antimicrobial medicines can be seen across both human and animal populations, linked to their increasing consumption for medical purposes of disease treatment [3] and their use as growth promoters in livestock husbandry and agriculture [4–6]. One cornerstone of global and national action plans to address AMR is to raise awareness [1]; however, focus on awareness building occurs at different priority levels in different country settings. Evidence on the best ways to reduce antibiotic use, and the impact of awareness raising activities is mixed [1,7–9]. A major focus of existing attempts has been on the ‘misuse’ of antibiotics for self-limiting conditions such as coughs, colds and viral upper-respiratory tract infections (URIs) [10,11]. This narrative centres on the ‘front line’ of medical practice; most notably healthcare professionals who prescribe and dispense antimicrobials such as antibiotics, as well as patients who are described as ‘demanding’ antimicrobials [12]. Across High-Income Countries (HICs) this ‘misuse’ is purported to reflect the hierarchy and habitus [13] of medical professionals [14,15] and their concerns over the safety of patients if antimicrobials are not prescribed [16]. In Low-Middle-Income Countries (LMICs) ‘non-judicious’ practices of antimicrobial prescribing and dispensing [17] have more often been assumed to reflect a lack of awareness of AMR and a lack of knowledge on the optimum use of antimicrobials, including antimalarials and antibiotics [18]. Despite being charged with irrational medicines use, there remains little empirical work that attends to what prescribers and dispensers across LMICs think, know, and do in relation to AMR and antimicrobials [19].

The dearth of information on how healthcare professionals prescribe and dispense antimicrobials across LMIC settings is an important research gap [20], and moreover the ways that this relates to awareness of AMR must be understood in context if successful strategies to quell AMR are to be developed and implemented [21–23]. This article presents findings from a multi-country study on AMR awareness and practice among human and animal healthcare professionals.

## Methods

### Ethics

This research was conducted in accordance with the Declaration of Helsinki and national and institutional standards. For each study site, ethical approval was obtained from the London School of Hygiene and Tropical Medicine and the relevant in-country ethics board. Ministry of Health and Human Services Kaduna State: MOH/ADM/744/VOL.1/539, LSHTM: 13717(Nigeria); The Liver Foundation Trust, LSHTM: 14048 (West Bengal Veterinary); Indian Centre for Media Studies Institutional Review Board: 1R800006230, LSHTM: 13696 (West Bengal Human); The Sierra Leone Ethics and Scientific Review Committee Freetown: letter dated 30 May 2017, LSHTM: 13807 (Sierra Leone); Addis Ababa Regional Health Bureau: A/A/H/B/7988/277, LSHTM: 13664 (Ethiopia); School of Pharmacy at Addis Ababa University (AAU), LSHTM: 13648 (Ethiopia); SRM Medical College Hospital and Research Centre: 1218/IEC/2017, LSHTM: 12262-1 (Chennai); WPRO 2017.16. PHL. 3. EMT, LSHTM: 13439 (Philippines); National Hospital for Tropical Diseases Scientific and Ethical Review Board, LSHTM: 13631 (Vietnam).

### Data collection and analysis

In-depth interviews and rapid ethnographic observation were undertaken with healthcare professionals in seven study sites across six LMICs, aiming to understand awareness of AMR and the relationships between awareness, context and antimicrobial use.

Five of the study sites included interviews with human practitioners only, two of the study sites included interviews with human and veterinary practitioners (Table 1).

**Table 1.** Samples across study sites

Country	Region	Type of healthcare practitioner	Number of participants interviewed
Vietnam	Hanoi	Human	24
Nigeria	Abuja	Human and animal	24
India	West Bengal	Human and animal	27
	Chennai	Human	30
Philippines	Manila	Human	61
Ethiopia	Addis Ababa	Human	67
Sierra Leone	Freetown	Human	11
**Total**	All	All	244

Inclusion criteria for the study required practitioners to be registered prescribers and/or dispensers of antimicrobial medicines. This research does not address AMR awareness and antimicrobial use among unregistered practitioners who are known to play a prominent role in the provision of antimicrobials across many LMIC settings [6]. The relevance of the research is therefore to the sections of healthcare services provided by trained healthcare professionals, whom we anticipate will have different and more standardised background training relevant to AMR and antimicrobials than the range of untrained providers in many settings.

Participants were selected using purposive sampling and subsequent snowball sampling aligned with the inclusion criteria. The sample aimed to include antimicrobial prescribing and/or dispensing doctors, nurses, dentists, pharmacists, medical educators and veterinarians. Research took place in urban or semi-urban settings; therefore, the study does not represent AMR awareness and antimicrobial practice by healthcare professionals in rural areas. All potential participants were invited to join the study through a written informed consent process which included permission to conduct, record and transcribe interviews. If participants declined to be recorded, with agreement the researcher took written notes. Interviews comprised the primary method of data collection, however researchers also carried out observations.

Each researcher was based with an in-country research institution (see ethical approval codes for names of institutions) who helped researchers identify and recruit potential participants. Researchers intended to interview as many participants as possible until saturation was reached. Constraints to recruitment included time constraints of researchers in identifying and scheduling interviews within a six-to-eight week duration, difficulty identifying large numbers of animal healthcare practitioners, and adverse weather conditions (monsoon/rainy season) which limited travel and negatively impacted interview schedules.

Researchers utilised a flexible participant-led approach to data collection, using pre-prepared questions, structured around a pre-determined set of topics as a guide, but trying where possible to follow the narrative of participants, attending to important contextual insights and not just to what individuals ‘knew’ about AMR.

Following collection, interview data were transcribed, and translated where relevant. Field notes were typed up from researcher observations. These materials were then subject to thematic analysis [24]. Transcripts were reviewed line by line, together with fieldnotes. Responses to core questions on awareness and knowledge were coded in a structured way to enable comparative analysis between respondents. The narrative from each respondent was then reviewed, to understand the ways they talked about resistance and antibiotic use in their practice. These narratives were then considered on a setting by setting basis, together with observation fieldnotes and photographs of the study context, in order to develop key themes for each setting. These themes were then revisited in a cross-setting analysis, involving dialogue with the primary data collectors as well as direct analysis of the materials, to draw out points of salience across settings and points of divergence. Four broad themes that emerged in this cross-setting analysis are explored in the results and discussion sections that follow. Quotes are anonymised and participant IDs reflect vocation (DXX doctor, DTXX dentist, NXX nurse, PXX pharmacist, EDXX educator, VXX Veterinarian). Data collection took place between May and July 2017. Data analysis was undertaken during July and August. Cross-setting analysis took place during September 2017.

## Results

In all, 244 healthcare professionals participated in the study including doctors (124) (general practice and surgical, hospital and community health facility based), pharmacists (70) (hospital and shop based), a dentist (1), nurses (3), medical educators (33) and veterinarians (13). The sample included professionals spanning the public and private sector, a boundary which appeared to be highly fluid, with many professionals working within both sectors either currently or previously. A majority were based in urban or peri-urban practices, reflecting the density of healthcare professionals in the selected countries. However, the aesthetic differences both across and within study sites were striking. From hospital doctors’ offices in West Bengal, lined with artworks gifted by medical representatives to nearby make-shift veterinary outhouses, tin fronted *ad hoc* pharmacy shops in the Philippines adorned with glass display cabinets of antimicrobials, hospital consultation rooms in Vietnam with bare metal bed frames and wall mounted fans attempting to circulate the humid air; the study sites captured the overwhelming material and phenomenological diversity in medical provision and practice all of which had profound implications, influencing how interviewees understood or displayed awareness of AMR. Interviewees ranged in age, included male and female participants and comprised medical trainees all the way through to late career professionals.

Taken together, the study sites indicate high levels of awareness of AMR and several cross-site recurring factors that influence antimicrobial use practices, including infrastructural issues of lacking diagnostics, availability of antimicrobials and shortages of medical staff, social issues such as affordability of antimicrobials, high levels of concern over poor hygiene and sanitation in both clinical and communal settings, and finally influential interactions with medical representatives that condition selection of antimicrobials.

### AMR awareness in practice

‘One of the problems we are facing in Nigeria, we use the broad-spectrum antibiotics, and so … it‘s not supposed to be like that, it‘s preferable, there‘s one article I read … WHO standard, that you are supposed to use a narrow-spectrum antibiotics… go for a specific antibiotics that will target that agent, but we go for broad-spectrum because these labs are not available.‘ V007 – Nigeria

‘I would rather over-treat and risk the resistance because there is indoor cooking the environment is not good there is malnutrition… so I would rather give antibiotics knowing that respiratory tract infection which is viral because I have no other way to treat.’ D008 – Ethiopia

Awareness of AMR across the study sites was high: almost all (99%) referred to resistance spontaneously or demonstrated knowledge of resistance in their responses. Most reported recognising AMR through treatment failure, for example symptoms failing to subside after antibiotic treatment or recurring illness episodes despite antibiotic treatment as indicative of resistance:

‘During the treatment we will come to know, for example lower generation antibiotic we are giving and still infection is not clearing, symptoms are still not suppressed then we will know that it may be resistance.’ ED006 – Chennai

Interviewees across the study sites also demonstrated knowledge of a variety of driving forces contributing to the growing prevalence of AMR, including the use of antibiotics for viral infections, inadequate dosing and duration of antibiotic medicines and the usage of substandard antibiotics – including contraband antibiotics and antibiotics used beyond their expiry date, or those stored in unsuitable conditions.

The way AMR awareness was enacted in practice included tailoring prescribing to anticipated resistance profiles within the limits of working contexts which included a lack of laboratory facilities for performing culture and sensitivity testing, a lack of antibiotics to choose from within the healthcare setting, and a shortage of medical staff. In some cases, being aware of potential resistance meant presumptive next-line therapy, particularly if the patient could afford to pay:

‘I have seen many cases here in Medicine department where meropenem is being given. Where the patient is suspected to have septicaemia there meropenem is being given. And giving macrolides like ceftriaxone, cefuroxime [sic – these antibiotics are classified as cephalosporins rather than macrolides] is most common in the wards. It is not like that they started from lower antibiotics because we know that lower antibiotics will not work because resistance has already been developed here and since the wards are so much dirty so it is quite obvious that lower antibiotics will not work here in this ward. So from the very beginning only we start from higher antibiotics.’ D019 – West Bengal

The absence of information about local resistance patterns was lamented and interviewees stated that this contributed to a disorganised and irregular way of constructing medical care in cases where patients did not respond to antimicrobial treatment. Respondents were keen to improve availability of guidelines on antimicrobial prescribing and dispensing that were relevant to their own setting, and the sensitivity profiles of bacteria in their settings.

### Pragmatic prescribing

‘If I prescribe Augmentin for them, I think I’m not fair to that individual, bearing in mind that there are a lot of adulterated, fake Augmentin in circulation so I looked around, what are the best ones that doesn’t (have) a lot of adulterated drugs and probably cheaper? I will look for the other one that’s a bit cheaper and usually it is not being adulterated… usually pharmaceutical doesn’t fake Septrin, so I will rather start with that Septrin.’ D002 – Nigeria

Tailoring prescribing according to potential resistance was one of many considerations for our participants. Both human and veterinary healthcare professionals related pragmatic practice in use of antibiotics, sensitive to economic, political and status issues relating to systems, patients and the medicines themselves. The lack of availability of antimicrobials led practitioners across the study sites to rely on a narrow set of antibiotics, prescribing and/or dispensing what was available in their setting. The type of antimicrobial selected was also dictated by perceived patient affordability. The idea of guiding treatment through diagnostic testing was also subject to economic evaluation. One doctor in Sierra Leone explained that even where such resources might be materially available, their unaffordability for patients precludes their use and thus reliance on antimicrobials as a cheaper and faster solution prevails:

‘The only constraint is sometimes the labs are very expensive, so you send a patient to a lab and they tell the patient, “Your test costs 250,000 Le[Leones],” the patient prefers to just go and get the drugs. Whether that is the condition or not, the patient still prefers the drug to the test. So, because of the cost, and sometimes delay. Some labs do delay with test results.‘ P001 – Sierra Leone

Concerns over the veracity of antibiotics led to preferences for particular brands of antibiotics over others, including antibiotics produced in Europe to those produced in China and the Middle East.

While veterinarians encountered similar issues to human healthcare professionals regarding availability, affordability and branding of antimicrobials, they also recounted specific concerns relating to livestock husbandry. A key concern was antibiotic withdrawal periods. One Nigerian vet, apprehensive about antibiotic residues in eggs for human consumption, described how he would select an antibiotic which might not be so effective in the eradication of disease, but that would have a shorter withdrawal period.

### Antimicrobials as care

‘It is frustrating. As a doctor, I am frustrated about it. I can’t provide the best care that I should be providing’ D003 – Sierra Leone

‘I have actually asked this question to my seniors, like you are putting the patient on meropenem right now without even considering other options, what if the patient needs another intervention later in his or her life… but their answer was like, I don’t care about what will happen in the future, right now we’re concerned about how to save our patient now… And they’re also correct in their regards, right, I mean we cannot really explain to the patient’s family that we killed their patients just because we were being very very cautious about the use of antibiotics, we cannot explain that.’ DO21 – West Bengal

A concern for both human and animal healthcare prescribers and dispensers across the study sites was the lack of available resources for providing care beyond the provision of antimicrobials. Reliance on antimicrobials as vehicles of care was a way to accommodate the overwhelming ratio of patients to healthcare practitioners, the lack of capacity and affordability of laboratory facilities, inadequate ward and clinic supplies, and unreliable patient care outside the clinical setting. These combined concerns were highlighted by a Nigerian doctor, who explains how these social, economic and political concerns determine antimicrobial use in the hospital setting:

‘We end up prescribing an empirical antibiotic… to avoid the progression of the disease, but this is not an efficient practice, but this is what we are forced to do because of the circumstances of our work environment, either because the facilities are overwhelmed by the number of patients, or the available staff on the ground are not enough.’ D003 – Nigeria

### Antimicrobials as hygiene

‘It’s overflowing with patients. People lie on the floor actually, we call them floor admissions. It is actually a term, ‘floor admission’ D021 – West Bengal

‘How can you practice sterile techniques and good hygiene practices when you have patients lying on the floor?’ D030-1 Philippines

Antimicrobials were prescribed and dispensed across the study sites as a replacement for poor hygiene and sanitation in both the community and the clinical setting that facilitated the contracting or spreading of disease. This spanned diverse concerns from open defecation in the community in West Bengal to cheap corrosive hand wash in a Vietnamese hospital which was purportedly reducing frequency of handwashing among staff. Across all study sites, inadequate facilities for patient isolation and ventilation in hospital settings encouraged reliance on antimicrobials to mitigate cross-infection within and between wards and patients. Human healthcare professionals also explained that concerns over high levels of malnutrition among the population, particularly in children, often led them to prescribe or dispense antimicrobials as a safety measure even where a disease episode was unlikely bacterial in nature. Hygiene and sanitation concerns were mirrored by veterinarians who stated that poor biosecurity in livestock rearing facilitated disease and conditioned reliance on antimicrobials.

### Commercial incentives

‘If (a) promoter comes and promotes one drug today it’s something that we see that for (a) certain number of days it’s only that drug that gets prescribed.’ P004 – Ethiopia (2)

Interactions with medical representatives were reported by human and animal healthcare providers across the study sites but were more commonly lamented by human healthcare professionals. Researchers observed the comings and goings of medical representatives during their observations particularly in clinical settings. These representatives were relied upon for up-to-date information on medicines, samples of medicines and opportunities for continuing professional development and profit. Healthcare professionals across the sites felt medical representatives exercised a high degree of influence over prescribing and dispensing practices and were concerned that their motives reflected business priorities rather than concern for patients or for the optimum use of medicines. The worrying nature of their influential practice is described here by a pharmacist in Chennai as motivating practitioners towards particular antibiotic medicines:

‘Representatives motivate doctors to prescribe more antibiotics and more expensive ones, higher generation too. Before doctors just used to give first generation, amoxicillin but now nobody prescribes basic antibiotics.‘ P002 – Chennai

## Discussion

This research aimed to understand awareness of antimicrobial resistance and how it related to antimicrobial use in nine settings in Ethiopia, India, Nigeria, Philippines, Sierra Leone and Vietnam. We found high levels of awareness of resistance, but this did not translate to hesitation to use antibiotics. Rather, we found evidence of escalation of antibiotic choice in a context of a lack of information on local resistance patterns. Prescribing and dispensing was moreover shaped by acute economic issues at the local level but also chronic infrastructural issues in health systems that have been built to rely on the presence of antimicrobials as a substitute for care and for hygiene. In this scenario, the role of pharmaceutical representatives is amplified, and their influence was palpable across settings. These findings suggest that simply increasing awareness of AMR will be insufficient to change prescribing and dispensing without local information on which antibiotics do work well, without investment in infrastructure that allows antimicrobials to be released from their ‘band aid’ role, and without active regulation of pharmaceutical representatives.

### AMR awareness: the knowledge behaviour conundrum

We found that while professionals were aware of AMR and the role of antimicrobial use in driving AMR, this knowledge did not translate into the desired practice of cautious antimicrobial use. In fact, we found evidence of awareness of AMR leading to escalated use of antimicrobials. This has clear implications for the potential development of resistance and needs to be considered in planning awareness raising campaigns. The reasons that awareness did not lead to desired practice was very clearly because numerous other factors affected prescribing and dispensing, in common across sites. These factors echo the research of many others who have demonstrated the influence of economic [25,26], infrastructural [6,27], commercial [6] and social factors [28] upon prescribing and dispensing practice and can be situated within wider anthropological discussions which posit antimicrobials as vehicles of care [29]. Healthcare practitioners across the study sites related their practice in ways that resemble ‘tinkering’ and ‘tailoring’ in the provision of medicines [30–32], which can be understood and problematised as rational strategies of care [30] within constrained environments, reflecting not a lack of awareness of AMR, but a lack of resources to adequately address AMR in daily practice [26,33,34].

Is this finding surprising? The weight of evidence listed above would suggest it should not be. But, the emphasis placed on raising awareness [1], and the continued mobilisation of the vocabulary of ‘irrational’ prescribing and dispensing by healthcare professionals, suggests that we continue to imagine a linear causal relationship between knowledge and practice [35], that can be fixed through provision of general education. Our reliance on these linear educational models continues in public health, despite studies highlighting why they routinely fail [36]. In the case of antimicrobial prescribing, scholars have shown that ‘knowledge may not be the clue’ [28] and highly publicised examples across topics such as tobacco [37] and HIV/AIDS [38] demonstrate that raising awareness or increasing knowledge often does not change practice in intended ways. Moreover, our finding that AMR awareness can actually affect prescribing in the opposite way to the intention of awareness programmes may be understood as counterintuitive, but it could also reflect the predictable reality that such campaigns are not received in a vacuum. The unintended consequences of public health campaigns have been described across topics [39,40] but are often overlooked in favour of a focus on desired effects. This has been understood as a facet of the current paradigm of evidence-based medicine that focuses narrowly on predetermined outcomes rather than evaluating more holistically ‘what happens’ [41]. This points to the need to go beyond the ‘empty vessel’ assumption that often underlies awareness campaigns [42]; in policy implementation it is important to recognise that awareness raising has the potential to do harm.

### Antimicrobials as band aids

Across LMIC settings, access to clean water, hygienic conditions for defecation, access to adequate nutrition and the resources to secure livestock from disease transmission remains sporadic and insecure, particularly in rural areas [6,43]. In the face of these threats to human and animal health, we found that antimicrobials were often deployed as a crucial band aid; prescribed and dispensed as a protective measure to mitigate the effects of health-compromising environments.

This finding underscores the importance of the link between hygiene and antimicrobial resistance, which has been highlighted by the WHO and the Inter-Agency Coordinating Committee [44]. However, initiatives to improve hygiene still tend to focus on behavioural strategies such as hand washing or cleaning, whereas our results highlight the infrastructural dimensions that antimicrobials are currently stop-gapping. It is clear from our findings that hygiene challenges emerge from issues beyond individuals’ control in their living and working conditions. Healthcare prescribers and dispensers across our study sites seem unlikely to change their practices until they feel more secure that the health and wellbeing of their patients and the animals of their clients will not be compromised as a result of their withholding antimicrobials. Policy that addresses infection prevention must address the infrastructural context of hygiene if it intends to impact prescribing practices.

### Medical representatives: big influence, little regulation

We are not the first to highlight the important role of medical representatives as a main purveyor of healthcare information in LMICs and as holding substantial influence over medical practice [45–48]. However, their roles are barely touched upon in global policy and national action plans on AMR. While medical and governmental institutions may have legislative checks or guidelines intended to regulate these relationships [48] it appears that such checks are largely absent in daily practice across many LMIC settings. The influence of these representatives points to a desire among practitioners for more information and better access to antimicrobials. Policy must address the need for information by local practitioners in regulatory frameworks if reliance on potentially unreliable profit orientated information sources is to be avoided. More research is vitally needed to explore not just the role and influence of medical representatives at the bottom end of the antimicrobial supply chain, but to interrogate further up the chain, acknowledging the multiple levels, agendas and motivations of the pharmaceutical industry.

### Limitations

The research focused on trained healthcare professionals and did not capture awareness of AMR or practices of antimicrobial prescribing and dispensing among unregistered healthcare providers. In many of our country settings, the majority of antimicrobial use is likely to occur among these providers. Our findings of high AMR awareness and the multiple other factors affecting practice may not resonate with these providers. Furthermore, only two of our study sites included veterinary practitioners and thus further research is required to understand whether our findings resonate with veterinary professionals in other settings. The question of generalisability is always a complex one for qualitative research. Here, generating generalisable results through purposive qualitative study is not achieved by having a representative sample but by collecting ideas from a wide range of people and interrogating these responses in a way that demonstrates points of similarity and points of divergence. Finally, pooling responses from qualitative interviews conducted in different country settings posed methodological challenges and showed that ‘awareness of AMR’ is often expressed and communicated differently across, as well as within, country settings. These challenges can be instructive as they show that we cannot take for granted the meaning of ‘awareness’ as a singular concept.

## Conclusion

The problem of rising antimicrobial resistance has been attributed to rising antimicrobial use in LMICs [20]. Healthcare professionals are often charged with ‘injudicious’ practice, which is often imagined to be a result of low levels of awareness of AMR and poor knowledge of the optimal use of antimicrobials. Our data challenges such assumptions, showing high AMR awareness among professionals across seven settings, and that rationales for everyday prescribing and dispensing practice reflect wider social, economic, investment and commercial factors. In pointing to the contingent and often precarious environments antimicrobials circulate in, this paper posits that reliance on antimicrobials by prescribers, dispensers, patients and clients, is unlikely to change until the settings in which antimicrobials remain suspended changes; until adequate resources for providing care and health facilitating environments for both human and animal populations are established.

## Paper context

Existing literature on AMR highlights irrational antimicrobial prescribing and dispensing and lack of awareness as crucial problems. There remains a dearth of research assessing awareness of AMR and contexts of antimicrobial usage. This paper shows high levels of awareness among healthcare professionals across LMIC settings which linked to the ready use of next-line antibiotics, and contextual factors that heavily influenced antimicrobial practice. Addressing contextual restraints will be as important as raising awareness in tackling AMR.
